# Deep-learning-based radiomics of intratumoral and peritumoral MRI images to predict the pathological features of adjuvant radiotherapy in early-stage cervical squamous cell carcinoma

**DOI:** 10.1186/s12905-024-03001-6

**Published:** 2024-03-19

**Authors:** Xue-Fang Zhang, Hong-yuan Wu, Xu-Wei Liang, Jia-Luo Chen, Jianpeng Li, Shihao Zhang, Zhigang Liu

**Affiliations:** 1 Radiotherapy department, Cancer center, The Tenth Affiliated Hospital, Southern Medical University(Dongguan People’s Hospital), No.78 Wandaonan Road, Dongguan, 523059 Guangdong People’s Republic of China; 2 Dongguan Key Laboratory of Precision Diagnosis and Treatment for Tumors, Dongguan, 523059 Guangdong People’s Republic of China; 3 Radiology Department, The Tenth Affiliated Hospital, Southern Medical University(Dongguan People’s Hospital), No.78 Wandaonan Road, Dongguan, 523059 Guangdong People’s Republic of China; 4 Pathology Department, The Tenth Affiliated Hospital, Southern Medical University(Dongguan People’s Hospital), No.78 Wandaonan Road, Dongguan, 523059 Guangdong People’s Republic of China

**Keywords:** Cervical squamous cell carcinoma, Radiomics, Magnetic resonance imaging, Deep learning, Adjuvant radiotherapy

## Abstract

**Background:**

Surgery combined with radiotherapy substantially escalates the likelihood of encountering complications in early-stage cervical squamous cell carcinoma(ESCSCC). We aimed to investigate the feasibility of Deep-learning-based radiomics of intratumoral and peritumoral MRI images to predict the pathological features of adjuvant radiotherapy in ESCSCC and minimize the occurrence of adverse events associated with the treatment.

**Methods:**

A dataset comprising MR images was obtained from 289 patients who underwent radical hysterectomy and pelvic lymph node dissection between January 2019 and April 2022. The dataset was randomly divided into two cohorts in a 4:1 ratio.The postoperative radiotherapy options were evaluated according to the Peter/Sedlis standard. We extracted clinical features, as well as intratumoral and peritumoral radiomic features, using the least absolute shrinkage and selection operator (LASSO) regression. We constructed the Clinical Signature (Clinic_Sig), Radiomics Signature (Rad_Sig) and the Deep Transformer Learning Signature (DTL_Sig). Additionally, we fused the Rad_Sig with the DTL_Sig to create the Deep Learning Radiomic Signature (DLR_Sig). We evaluated the prediction performance of the models using the Area Under the Curve (AUC), calibration curve, and Decision Curve Analysis (DCA).

**Results:**

The DLR_Sig showed a high level of accuracy and predictive capability, as demonstrated by the area under the curve (AUC) of 0.98(95% CI: 0.97–0.99) for the training cohort and 0.79(95% CI: 0.67–0.90) for the test cohort. In addition, the Hosmer-Lemeshow test, which provided *p*-values of 0.87 for the training cohort and 0.15 for the test cohort, respectively, indicated a good fit. DeLong test showed that the predictive effectiveness of DLR_Sig was significantly better than that of the Clinic_Sig(*P* < 0.05 both the training and test cohorts). The calibration plot of DLR_Sig indicated excellent consistency between the actual and predicted probabilities, while the DCA curve demonstrating greater clinical utility for predicting the pathological features for adjuvant radiotherapy.

**Conclusion:**

DLR_Sig based on intratumoral and peritumoral MRI images has the potential to preoperatively predict the pathological features of adjuvant radiotherapy in early-stage cervical squamous cell carcinoma (ESCSCC).

**Supplementary Information:**

The online version contains supplementary material available at 10.1186/s12905-024-03001-6.

## Introduction

Cervical cancer ranks fourth in prevalence among women globally and is the predominant gynecologic malignancy globally [1]. With the implementation of cervical cancer screening programs, a growing number of early-stage cervical cancer patients, categorized as stage IA to IIA according to The International Federation of Gynecology and Obstetrics (FIGO) classification, are being effectively managed. Surgical intervention is a viable option for these patients, although 20% may experience fatal pelvic recurrence [2]. Concurrent adjuvant radiochemotherapy significantly improved the 4-year Progression-Free Survival (PFS) from 63 to 80% and elevated the 4-year Overall Survival (OS) from 71 to 81% among high-risk groups based on the Peter Standard with histologically confirmed positive pelvic lymph nodes, positive parametrial involvement, and/or positive surgical margin [3]. Studies GOG 49 and GOG 92 have demonstrated that factors such as depth of tumor invasion (DOI), periuterine involvement, Lymphovascular Space Invasion(LVSI), tumor grade, and gross tumors have a significant impact on the risk of recurrence in cervical squamous cell carcinoma(CSCC); [4] Subsequently, the Sedlis standard was introduced, reducing the recurrence rates from 27.9 to 15.3% [5]. Postoperative radiotherapy is recommended for patients with early-stage cervical squamous cell carcinoma(ESCSCC) if their postoperative pathological features meet the Peter or Sedlis standard [6, 7]. A randomized study demonstrated that both radical surgery and radiotherapy yield comparable effectiveness in the treatment of ESCSCC [8]. The combination of surgery and radiotherapy significantly heightens the risk of complications, including lower limb edema and urinary system adverse events [2, 8]. Utilizing a biomarker, predicting the patients with high-risk pathological features for adjuvant radiotherapy before surgery, is crucial for selecting the most suitable therapy for each patient, thereby ensuring optimal treatment outcomes with minimal complications.

Recent studies have demonstrated significant advancements in the application of artificial intelligence methods to the processing of medical images within the medical field, particularly in the diagnosis and treatment of malignant tumors [9–12]. Magnetic resonance imaging (MRI) is the optimal radiological approach for evaluating primary tumors larger than 10 mm in size [13]. Recent studies on MRI radiomics have demonstrated advancements in predicting the tumor grade, DOI, LVSI, pelvic lymph node metastasis (LNM) and other postoperative pathological features in early-stage cervical cancer [14–20]. In comparison to radiomics, deep-learning networks, such as convolutional neural networks, can be trained using end-to-end supervised methods. Simultaneously, it can learn highly distinctive image features and mitigate the influence of human judgment on radiological features [21]. It offers a higher degree of flexibility and can be integrated into intricate models [22]. 

We hypothesized that MRI images of the intratumoral and peritumoral regions are indicative of the pathological features for postoperative adjuvant radiotherapy in patients with ESCSCC. This study aims to investigate the feasibility of a Deep-Learning Radiomic Signature using intratumoral and peritumoral MRI images in order to predict the pathological features relevant to postoperative adjuvant radiotherapy for ESCSCC.

## Materials and methods

### Study population

This retrospective study was approved by the Ethics Committee of The Tenth Affiliated Hospital, Southern Medical University(Dongguan People’s Hospital) (KYKT2022-062).

A total of 289 patients who underwent radical hysterectomy and pelvic lymph node dissection during the period from January 2019 to April 2022 at The Tenth Affiliated Hospital, Southern Medical University(Dongguan People’s Hospital) were included in the study. These patients had complete MRI scans and were classified as stage IB1-IIB based on the 2018 FIGO staging system. The inclusion criteria for the study were as follows: (1) patients who underwent a pelvic MRI examination within seven days prior to surgery, (2) availability of pathologic evaluation for variables including DOI, LVSI, LNM, tumor size, surgical margin, and parametrium, (3) confirmation of postoperative pathology showing squamous cell carcinoma (SCC), and (4) radical hysterectomy covering a minimum of the upper 3 to 4 cm of the vaginal cuff, parametria, and adjacent nodal basins (such as the external and internal iliac, obturator, and presacral nodes). Exclusion criteria for the study were as follows: (1) patients who received neoadjuvant chemotherapy, radiotherapy, or cervical conization treatment prior to surgery, (2) patients with tumors that were not visible on sagittal T2WI, due to the difficulties in accurately defining regions of interest (ROIs) for such tumors, (3) insufficient image quality for the extraction of radiomics features, and (4) patients who were either breastfeeding or pregnant. All patients were randomly divided into the training and validation cohorts with a 4:1 ratio.

**Fig. 1:**
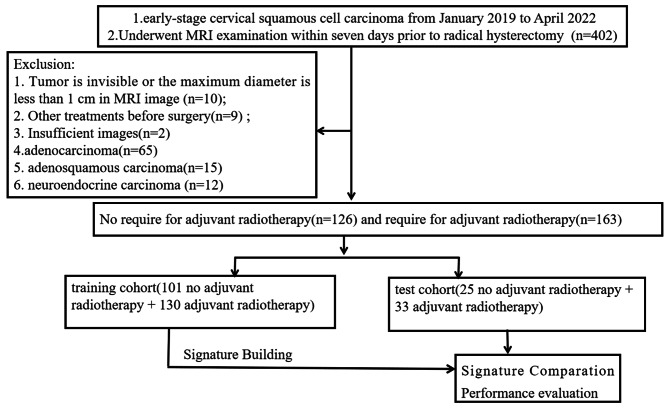
Flow chart of the patient recruitment process

A flowchart of this study is presented in Fig. 1.

We obtained clinical data from medical records, which consisted of information such as age, tumor size measured using MRI (referred to as maximal tumor diameter, MTD, on MR images of the largest lesions), presence of lymph node involvement (identified through MRI), presence of vaginal involvement (detected through MRI), parametrial invasion (assessed clinically through palpation), and serum levels of various markers including squamous cell carcinoma antigen (SCC-Ag), white blood cells, neutrophils, lymphocytes, monocytes, erythrocytes, hemoglobin, thrombocytes, alkaline phosphatase (ALP), lactate dehydrogenase (LDH), albumin (ALb), creatinine, lymphocyte/monocyte ratio (LMR), and lymphocyte/neutrophil ratio (LNR). The need for adjuvant radiotherapy after surgery is mainly determined by postoperative pathology and the clinical examination before surgery. Adjuvant radiotherapy is recommended as long as Peter’s standards are met: surgical margin, parametrium and lymph node. Cases that are negative for lymph node, margin, parametria comply with Sedlis’ standards, which are commonly utilized for determining postoperative treatment for ESCSCC: presence of LVSI, tumor invasion extending to the deep 1/3 of the stromal depth with any tumor size; presence of LVSI, tumor invasion extending to the middle 1/3 of the stromal depth with a tumor size of 2 cm or larger; presence of LVSI, tumor invasion extending to the superficial 1/3 of the stromal depth with a tumor size of 5 cm or larger; absence of LVSI, tumor invasion extending to the deep or middle 1/3 of the stromal depth with a tumor size of 4 cm or larger.

### MRI image acquisition protocol

All patients enrolled in the study underwent pelvic MRI using either an 18-channel abdominal-phased array coil with a 3.0T MRI scanner (Skyra, Siemens, Germany, *N* = 93, 32.18%) or a 1.5T-MRI scanner (Skyra, Siemens, Germany, *N* = 289, 67.82%). The MRI protocols employed included T1-weighted imaging (T1WI), T2-weighted imaging (T2WI), T2-weighted imaging with fat suppression (T2fs), and contrast-enhanced T1-weighted imaging (CE-T1) in axial views. The imaging parameters comprised a layer thickness of 5 mm, a layer interval of 1.0 mm, a matrix size of 384 × 320, and a field of view (FOV) of 360 mm. Following sequence scanning, a contrast medium of 0.1 mmol/kg was injected intravenously via the elbow at an injection speed of 3 mL/s. Subsequently, enhanced scanning was performed in axial views using a 3D-SPGR sequence with slice thicknesses of 3.0 mm and slice intervals of 1.0 mm.

### Data preprocessing

Under different imaging panels, the range of pixel values of medical images varies significantly. To reduce the side-effect of pixel value outliers, we sorted all the pixel values in each image and truncated the intensities to the range of 0.5 to 99.5 percentiles.

Regions of interest (ROI) are common with heterogeneous voxel spacing because of different scanners or different acquisition protocols. Such spacing refers to the physical distance between two pixels in an image. Spatial normalization reduces the effect of voxel spacing variation. In our experiment, we utilized a fixed resolution resampling method to address the these challenges.

### Development of the ROI

The regions of interest (ROI) were manually delineated slice by slice on the ITK-snap software (version 3.0.0, www.itk-snap.org) by two radiation oncology specialists with over 10 years of experience. A radiologist with over 15 years of experience validated the manual delineations.The delineation of ROIs is stored in the NII format as a mask for subsequent analysis.

The original fragments of ROIs are expanded at one-voxel intervals outside the tumor using the onekey platform.

### Radiomics procedure

#### Feature extraction

The handcrafted features were divided into three groups: geometry, intensity and texture. We employed geometry to describe the three-dimensional shape characteristics, intensity to describe the first-order statistical distribution of voxel intensities, and texture to describe other features such as patterns, as well as the second- and high-order spatial distributions of intensities. Various methods, such as the gray-level co-occurrence matrix (GLCM), gray-level run-length matrix (GLRLM), gray-level size-zone matrix (GLSZM), and gray-level dependence matrix (GLDM), were utilized to extract the texture features.

We obtained radiomics data from six modalities, including MRI images (CE T1, T2WI, T2fs) of both intratumoral and peritumoral regions. We utilized the early fusion method to combine all radiomics features from different modalities, and then fed them into the subsequent process.

#### Feature selection

We conducted a test-retest analysis to evaluate the reliability. Twenty patients were randomly selected from the dataset, and the tumor subregions of each patient were segmented twice by a specialist. Additionally, the ROI subregions of each patient were independently segmented by two radiation oncology specialists.The features extracted from these multiple-segmented subregions were evaluated using the intraclass correlation coefficient (ICC). Features with an ICC greater than 0.85 were considered to demonstrate favorable reproducibility and were selected for further analysis.

Mann-Whitney U test and feature screening were performed for all radiomic features. We retained only features with a *p*-value less than 0.05. Spearman’s rank correlation coefficient was also used to assess the correlation between features, and we kept only one of the features when the correlation coefficient exceeded 0.9. To ensure the most comprehensive depiction of features, we employed a greedy recursive deletion strategy for feature filtering. This involved removing the feature with the highest redundancy in the current set every time it was used. Adjusting regulation weights λ, LASSO shrunk all regression coefficients to zero and accurately set the coefficients of unrelated features to zero. We employed the 10-fold cross validation with minimum standards to find the optimal λ, the final value that generated the minimum cross validation error. The retained non-zero coefficient features were combined and used to create a radiomics signature through regression model fitting. Next, we gained the radiomics score for each patient through a linear combination of preserved features that were weighted using their model coefficients. We conducted the Python scikit-learn package provided by the onekey-platform for LASSO regression modeling.

#### Deep learning procedure

In our study, four pre-trained CNN models (resnet50, resnet101, inception_v3, densenet121) were specifically chosen for their effectiveness in handling the ILSVRC-2012 dataset. We chose the slice that exhibited the largest tumor area to represent each patient. We used min-max transformation to normalize the gray values to range [-1, 1]. Next, we cropped each subregion image, resizing to 224 × 224 with nearest interpolation. The obtained images can be used as the model input.

To mitigate data leakage in the image data, we meticulously adjusted the learning rate to enhance generalization. The cosine decay learning rate algorithm was implemented for this purpose. The specific learning rate utilized is presented below:

$${\eta }_{t}^{task-spec}={\eta }_{min}^{i}+\frac{1}{2}\left({\eta }_{max}^{i}-{\eta }_{min}^{i}\right)\left(1+cos\left(\frac{{T}_{cur}}{{T}_{i}}\pi \right)\right)$$

${\eta }_{min}^{i}=0$, ${\eta }_{max}^{i}$ =0.01, ${T}_{i}=50$ refers to the minimum learning rate, the maximum learning rate, and the number of iteration epochs, respectively. Because the backbone takes pre training parameters, in order to ensure the migration effect, we used ${T}_{cur}=\frac{1}{2}{T}_{i}$to fine tune the parameters of the backbone part. Therefore, the backbone part’s learning rate is as follows:

$${\eta }_{t}^{backbone}=\left\{\begin{array}{ll}0& \text{ if }{T}_{cur}\le \frac{1}{2}{T}_{i}\\ {\eta }_{min}^{i}+\frac{1}{2}\left({\eta }_{max}^{i}-{\eta }_{min}^{i}\right)\left(1+cos\left(\frac{{T}_{cur}}{{T}_{i}}\pi \right)\right)& \text{ if }{T}_{cur}>\frac{1}{2}{T}_{i}\end{array}\right.$$

Other hyperparameter configurations included: optimizer: SGD, loss function: sigmoid cross entropy.

#### Signature building

##### Clinical/Radiomics Signature

After conducting Lasso feature selection, we fed the final features into MLP machine-learning models to build risk models. To obtain the final Clinic/Rad Signature, we employed 5-fold cross-validation in this study.

##### Deep Transformer Learning Signature

Among the four models, ResNet101 model has the best predictive performance (Supplementary Table 2). We utilized the ResNet101 model as the base model for our Deep Transfer Learning Signature (DTL_Sig) to estimate the probability of each sample. To obtain the final prediction, we employed a fusion method called stacking. Specifically, we trained each model individually and then utilized the late fusion method to evaluate deep learning as features. Finally, we utilized MLP to generate the final prediction.

##### Deep-Learning Radiomic Signature

In the process of constructing the Deep Learning Radiomics Signature (DLR_Sig), we combined the Radiomics Signature and Deep Transformer Learning Signature using a logistic regression model.

A five-fold cross-validation method was employed, and the test cohort was fixed to ensure fair comparisons. To assess the efficiency of each signature, we selected the best model from the construction process of each signature.

#### Statistical analysis

Receiver Operating Characteristic (ROC) curves were plotted to evaluate the diagnostic performance of the signatures in both the training and test cohorts. The ROC of these models were compared using the DeLong test [23]. Calibration curves and the Hosmer-Lemeshow analytical fit were used to assess the calibration performance of the signatures. We employed the Mapping decision curve analysis (DCA) to evaluate the clinical utility of the four signatures.

All data analyses were conducted using Python 3.7.12 on the OnekeyAI platform 3.1.8. For statistical analyses, we used statsmodels version 0.13.2. The χ² test was utilized for discrete variables, while the independent sample t-test was applied to continuous variables to compare the clinical characteristics of patients. The pyradiomics package version 3.0.1 was used to extract radiomics features. Machine learning algorithms such as support vector machines (SVMs) were implemented using the scikit-learn package version 1.0.2. Additionally, deep learning models were developed based on torch version 1.11.0, utilizing cuda 11.3.1 and cudnn8.2.1. *P*-values < 0.05 were considered statistically.

**Fig. 2:**
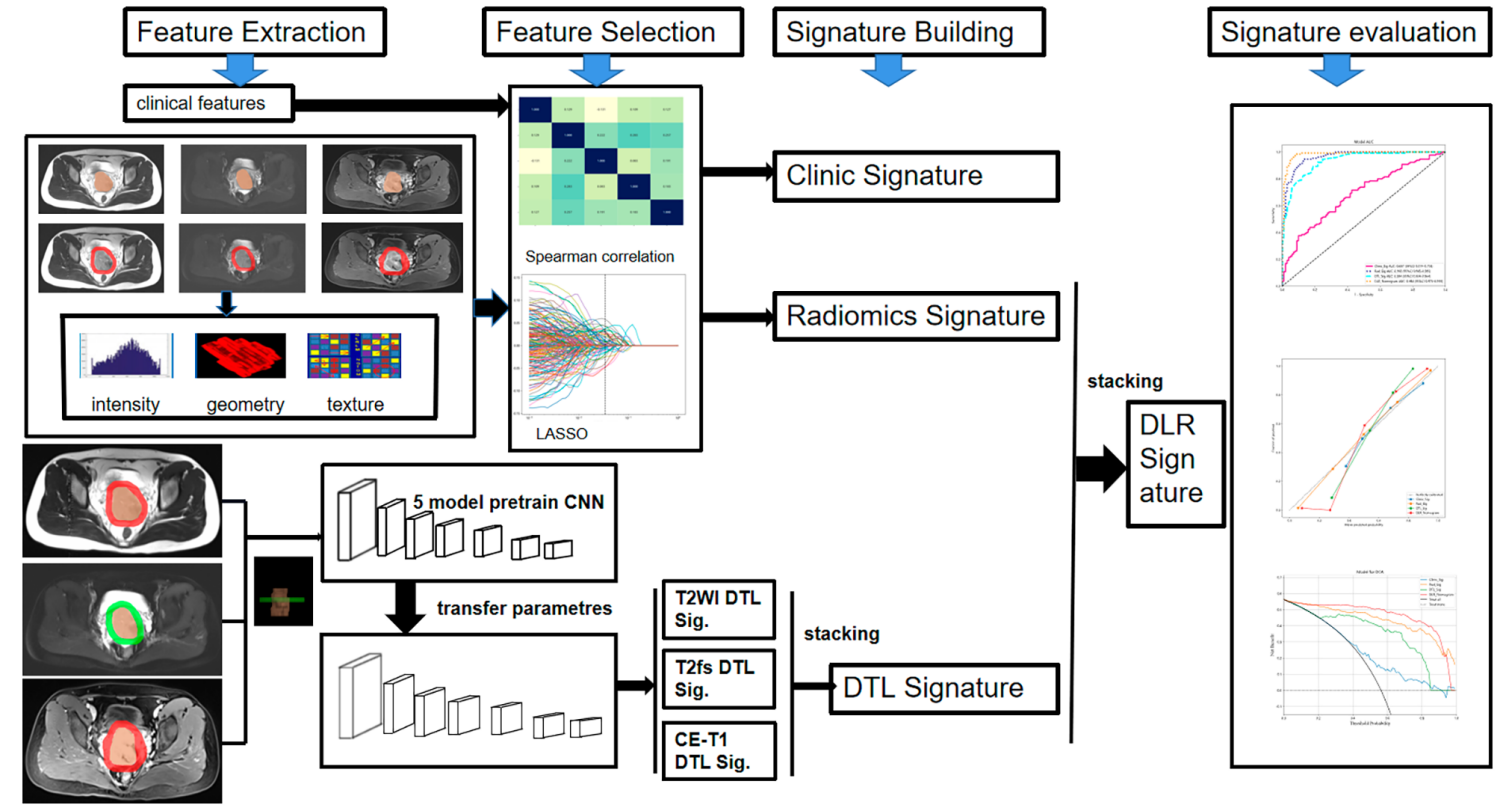
Schematic illustration of the Signature Building and Comparation

The progress of signature building and comparison was displayed in Fig. 2.

## Results

### Feature statistics

#### Clinical features statistics

The patients had an average age of 49.30 years, with an average MRI tumor size (MTD) of 3.24 cm. Among the patients, 112 (38.75%) were positive for vaginal involvement, 43 (14.88%) were positive for pelvic lymph node involvement through MRI, and 17 (5.88%) showed parauterine invasion through clinical palpation before surgery. There were significant differences in the distribution of age (*p* = 0.03), SCC-Ag (*p* < 0.001), MTD-MRI (*p* < 0.001), and Lymph node-positive MRI (*p* < 0.001) between the adjuvant and non-adjuvant radiotherapy groups. No significant differences were found in other characteristics between the two groups, including vaginal involvement (MRI), parametrial invasion (clinical palpation), and serum levels of white blood cells, neutrophils, lymphocyte, monocytes, erythrocytes, hemoglobin, thrombocytes, ALP, LDH, ALb, creatinine, LMR, and LNR (Table 1). As displayed in Table 1, the clinical characteristics in the training and test groups were balanced, and there was no statistical difference between the two groups.

**Table 1:** Clinical features of patients in training cohort and test cohort

Variable	All cohort	Training cohort			Test cohort			*P*-value*	*P*-value**
		no AR (*n* = 101)	AR(*n* = 130)	*P*	no AR (*n* = 25)	AR (*n* = 33)	*P*-value		
Age(year)***	49.30 ± 10.16	48.64 ± 11.14	50.58 ± 9.38	0.10	45.52 ± 8.56	49.12 ± 10.59	0.15	0.13	0.03
parametrial invasion-clinical palpation				0.15			1.00	1.00	0.14
No ( n, % )	272 (94.12)	98(97.03)	119(91.54)		24(96.00)	31(93.94)			
Yes ( n,% )	17 (5.88)	3(2.97)	11(8.46)		1(4.00)	2(6.06)			
MTD-MRI(cm)	3.24 ± 1.41	2.85 ± 1.34	3.45 ± 1.33	< 0.001	2.84 ± 1.42	3.88 ± 1.53	0.02	0.21	< 0.001
vagina-positive MRI				0.79			0.53	0.76	1.00
No ( n,% )	177 (61.25)	64(63.37)	79(60.77)		13(52.00)	21(63.64)			
Yes ( n,% )	112 (38.75)	37(36.63)	51(39.23)		12(48.00)	12(36.36)			
Lymph node-positive MRI				0.01			0.13	0.44	< 0.001
No ( n,% )	246 (85.12)	94(93.07)	105(80.77)		23(92.00)	24(72.73)			
Yes ( n,% )	43 (14.88)	7(6.93)	25(19.23)		2(8.00)	9(27.27)			
MRI scanner				0.78			1.00	0.56	0.79
3.0 T ( n,% )	93(32.18)	30(29.70)	42(32.31)		9(36.00)	12(36.36)			
1.5 T ( n,% )	196(67.82)	71(70.30)	88(67.69)		16(64.00)	21(63.64)			
White blood cell(×10^9^/L)***	9.94 ± 3.46	9.96 ± 3.40	10.08 ± 3.05	0.57	10.05 ± 3.88	9.23 ± 4.68	0.49	0.63	0.92
Neurtophil(×10^9^/L)***	8.01 ± 3.41	7.99 ± 3.49	8.07 ± 3.06	0.63	8.27 ± 3.80	7.68 ± 4.20	0.56	0.87	0.89
Lymphocyte(×10^9^/L)***	1.31 ± 0.56	1.32 ± 0.59	1.37 ± 0.59	0.55	1.21 ± 0.44	1.15 ± 0.34	0.48	0.10	0.77
Erythrocytes(×10^12^/L)***	3.69 ± 0.66	3.77 ± 0.59	3.75 ± 0.59	0.73	3.52 ± 0.65	3.29 ± 0.93	0.15	< 0.001	0.41
Hemoglobin(G/L)***	104.68 ± 18.45	106.55 ± 16.71	105.32 ± 17.08	0.44	105.72 ± 11.45	95.64 ± 28.61	0.09	0.23	0.17
Thrombocyte(×10^9^/L)***	220.58 ± 70.43	217.62 ± 62.25	233.42 ± 72.41	0.11	202.28 ± 60.00	192.97 ± 83.40	0.77	0.05	0.21
ALP(U/L)***	53.76 ± 39.09	49.97 ± 39.92	59.08 ± 39.32	0.11	49.96 ± 31.81	47.33 ± 39.60	0.9	0.21	0.15
LDH(U/L)***	121.56 ± 82.38	113.15 ± 85.56	130.84 ± 79.93	0.12	125.62 ± 76.71	107.67 ± 85.11	0.59	0.59	0.26
ALb(G/L)***	28.14 ± 17.78	26.69 ± 18.91	29.57 ± 16.66	0.86	30.41 ± 17.16	25.23 ± 18.97	0.37	0.78	0.80
Creatinine(mmol/L)***	30.62 ± 29.57	25.49 ± 29.13	35.27 ± 29.68	0.03	37.32 ± 28.54	22.95 ± 28.06	0.07	0.72	0.23
SCC-Ag(ng/mL)***	3.45 ± 6.51	1.86 ± 2.41	4.86 ± 8.67	< 0.001	2.10 ± 1.96	3.82 ± 6.30	0.92	0.47	< 0.001
Mononuclear cell(×10^9^/L)***	0.52 ± 0.28	0.52 ± 0.22	0.52 ± 0.34	0.27	0.50 ± 0.19	0.54 ± 0.27	0.97	0.73	0.30
LMR***	2.96 ± 1.71	2.84 ± 1.39	3.20 ± 2.03	0.42	2.75 ± 1.42	2.58 ± 1.31	0.63	0.21	0.62
LNR***	0.23 ± 0.20	0.22 ± 0.19	0.22 ± 0.19	0.89	0.22 ± 0.22	0.26 ± 0.27	0.73	0.88	0.80

AR: adjuvant radiotherapy; MTD-MRI:maximal tumor diameter on MR images of the largest lesions; ALP: alkaline phosphatase; LDH: lactate dehydrogenase ALb: albumin; SCC-Ag: squamous cell carcinoma antigen; LMR: lymphocyte/monocyte ratio; LNR: lymphocyte/neutrophil ratio;*P*-value*: the difference of each clinical variable between the training and test cohorts; *P*-value**: the difference of each clinical variable between no adjuvant radiotherapy group and adjuvant radiotherapy group. *P* < 0.05 indicates statistically significant. ***: mean, standard deviation.

No linear relationship exists between these clinical features. The Spearman correlation coefficients between the four features and postoperative adjuvant radiotherapy are illustrated in Supplementary Fig. 1.

#### Radiomics features statistics

A total of 84 shape features, 2160 first-order features, and 8160 texture features were extracted(Supplemental Fig. 2). The shape features were described using geometry, while the first-order features were described using intensity measures, specifically: gray-level co-occurrence matrix (GLCM), gray-level run length matrix (GLRLM), gray level size zone matrix (GLSZM), and Gray Level Dependence Matrix (GLDM). We extracted all handcrafted features using a custom feature analysis program implemented in Pyradiomics.(http://pyradiomics.readthedocs.io).

**Fig. 3:**
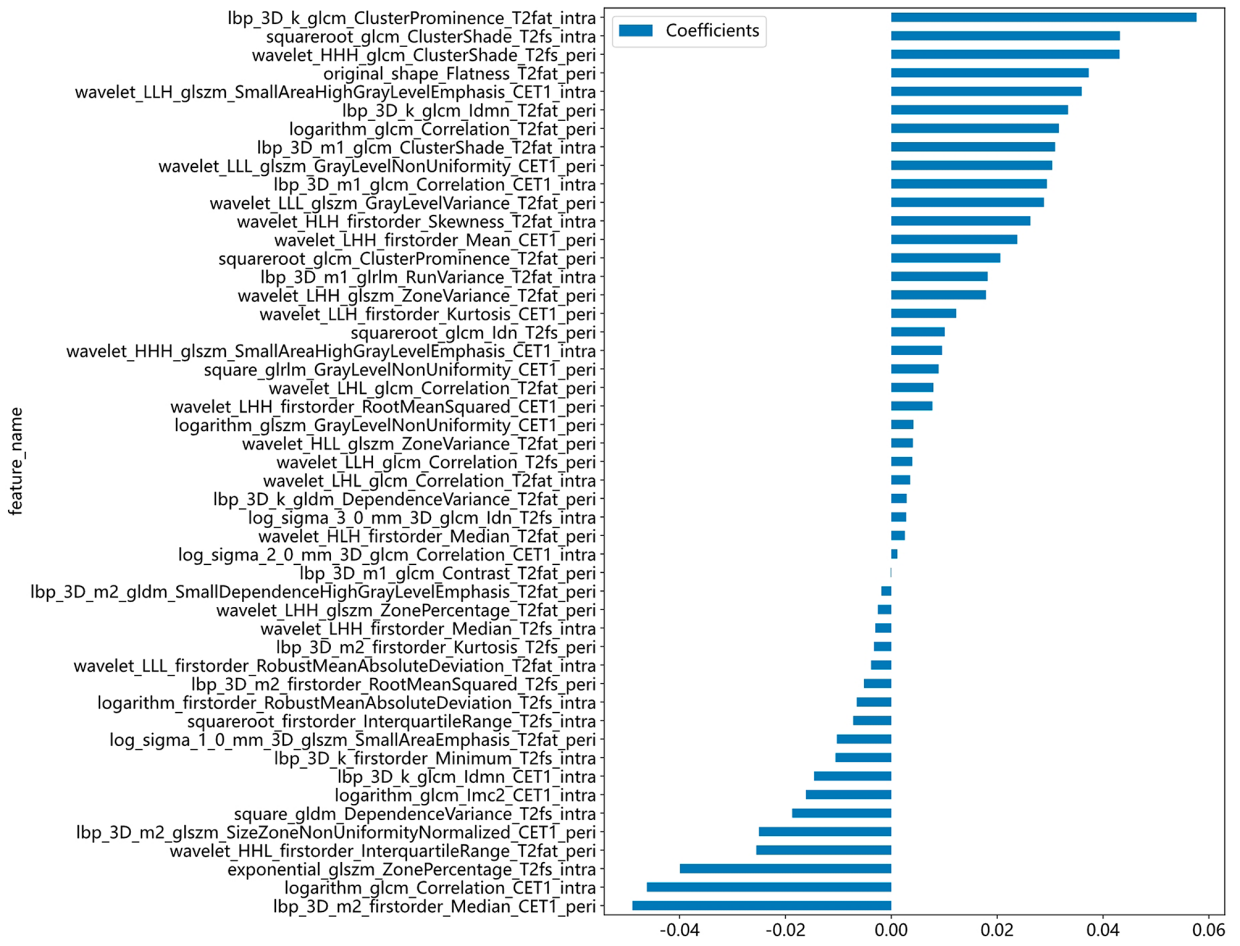
The histogram of the Radiomics Signature based on the selected features

Finally, a total of 49 features of non-zero coefficients were selected to establish the Rad-score with a LASSO logistic regression model, including 21 intratumoral features, 28 peritumoral features, 15 features of CE-T1 sequences, 21 features of T2WI sequences, and 13 features of T2fs sequences. The number and ratio of each group of handcrafted features, all features and corresponding *p*-value results, coefficients of 10-fold cross validation, MSE (mean standard error) of 10-fold cross validation were displayed in Supplemental Fig. 3. The histogram of the Rad_Sig based on the selected features is displayed in Fig. 3.

#### Deep transformer learning signature

We employed the Resnet101 model for the DTL_Sig, which focused on CE T1, T2WI, T2fs within the peritumoral ROI. Among these, modal T2WI achieved the highest AUC of 0.76 (95%CI: 0.64–0.89) in our test cohort (Supplemental Table 2).

### Signature comparation

**Fig. 4:**
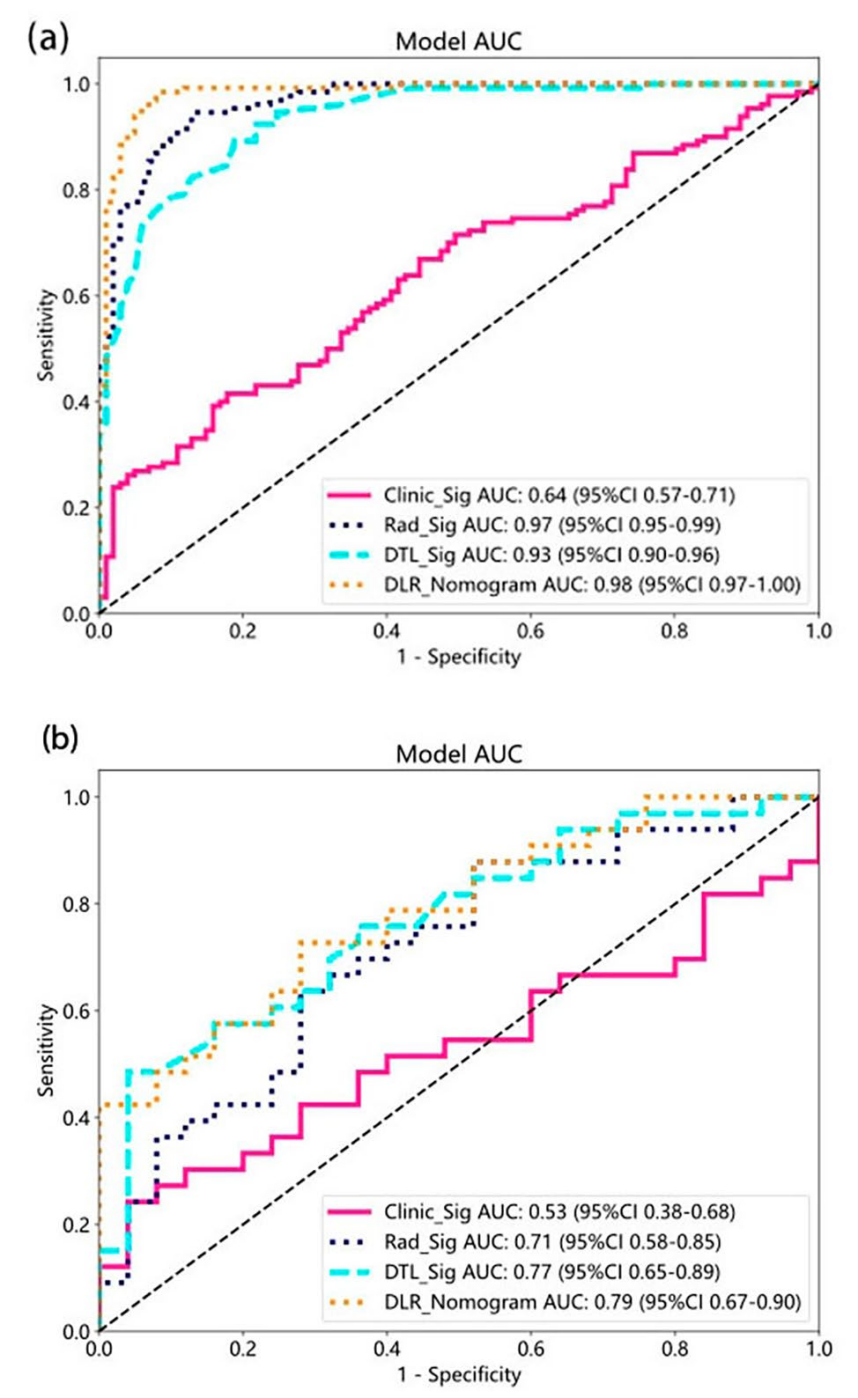
The AUCs of different signatures on the training **(a)**//test cohort **(b)**

**Fig. 5:**
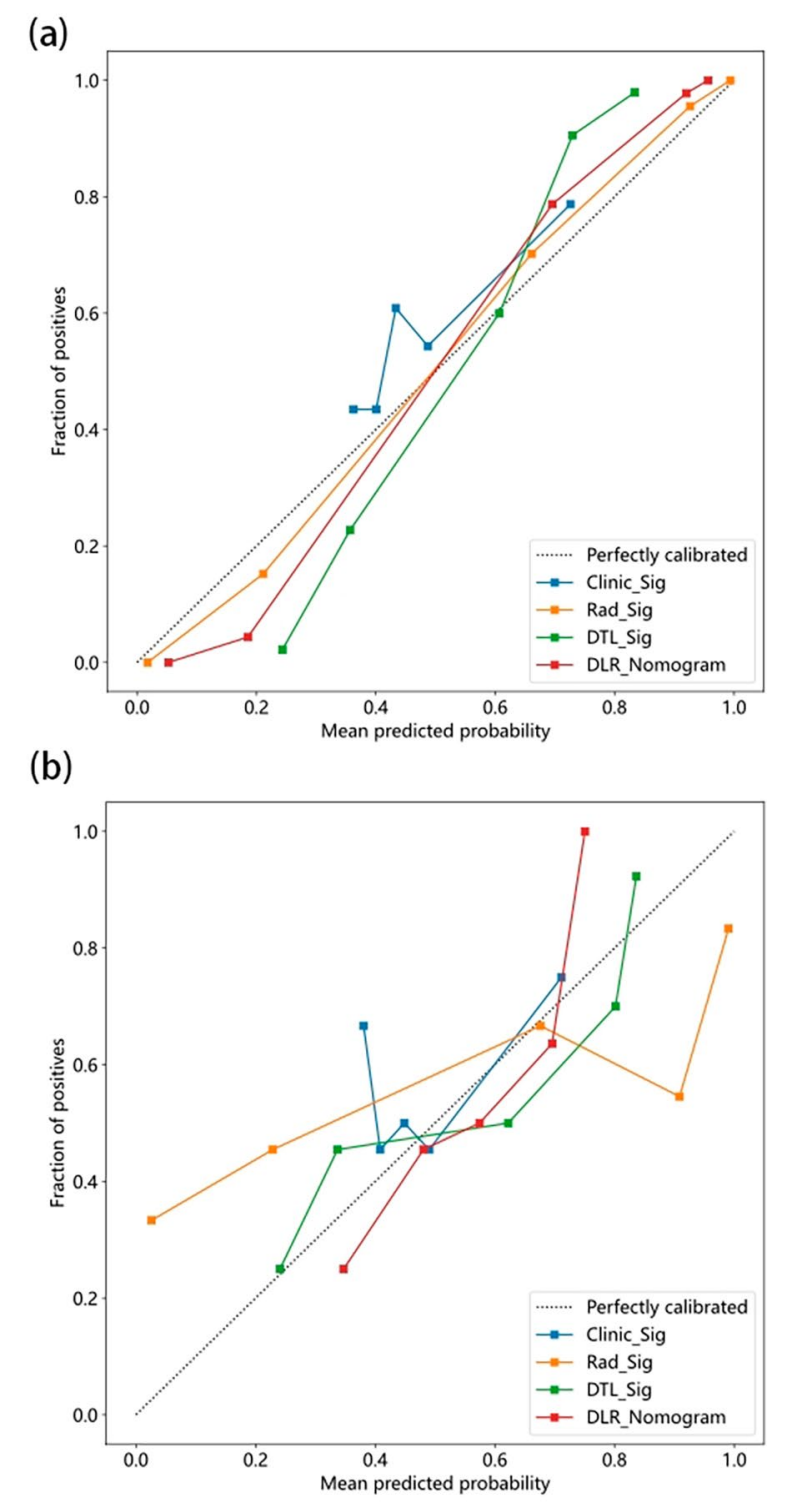
The calibration curves of different signatures on the training**(a)**/test cohort **(b)**

**Fig. 6:**
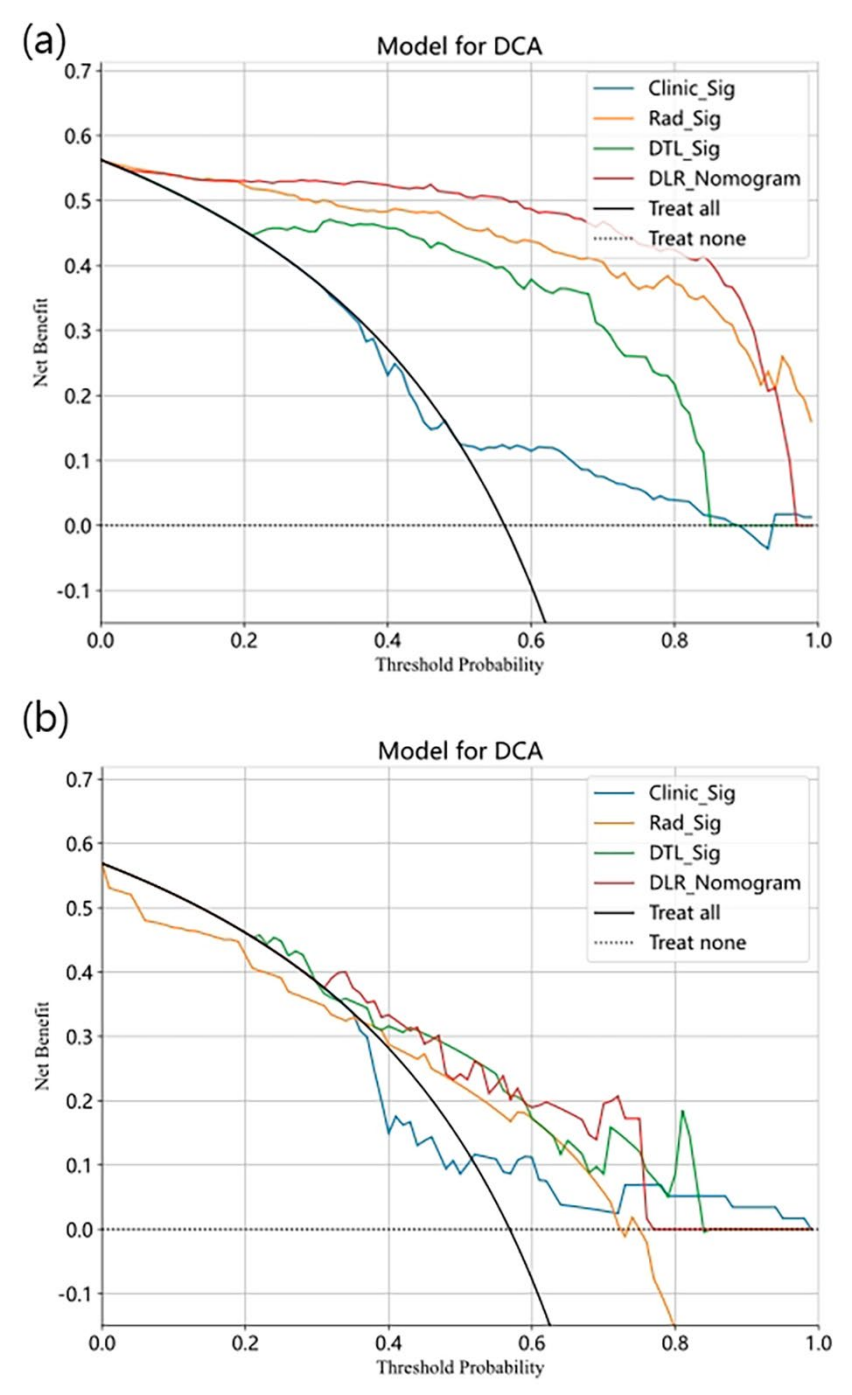
The decision curves of different signatures on the training**(a)**/test cohort **(b)**

DLR_Sig achieved an AUC of 0.98 (95% CI: 0.97-1.00) in the training cohort and 0.79 (95% CI: 0.67–0.90) in the test cohort. DTL_Sig obtained AUCs of 0.93 (95% CI: 0.90–0.96) in the training cohort and 0.77 (95% CI: 0.65–0.89) in the test cohort. Rad_Sig yielded AUCs of 0.97 (95% CI: 0.95–0.99) in the training cohort and 0.71 (95% CI: 0.58–0.85) in the test cohort. Clinic_Sig yielded AUCs of 0.64 (95% CI: 0.57–0.71) in the training cohort and 0.53 (95% CI: 0.38–0.68) in the test cohort. DLR_Sig demonstrates the highest efficiency in predicting adjuvant radiotherapy (see Fig. 4). According to Table 2, the *p*-values of the DeLong test between Clinic_Sig and the other models were all less than 0.05, indicating a significant difference in predictive performance between Clinic_Sig and the other models in the training/test cohort. The above AUCs indicate that Rad_Sig, DTL_Sig, and DLR_Sig are more effective at predicting pathological features of adjuvant radiotherapy than Clinic_Sig. The DeLong test showed that there is no significant difference among the predictive effectiveness of Rad_Sig, DTL_Sig, and DLR_Sig, although the AUC values of these models gradually improve. Figure 5 presents the calibration of the four signatures. Specifically, the calibration plot of the DLR_Sig is very close to the ideal curve, indicating excellent consistency between the actual and predicted probabilities of the pathological features of adjuvant radiotherapy. The DLR_Sig displayed excellent calibration, as demonstrated by the non-significant *p*-values of 0.87 in the training cohort and 0.15 in the test cohort according to the Hosmer-Lemeshow test. Figure 6 displays the decision curve analysis (DCA) for the four models. The DCA curve indicates that using the DTL_Sig to predict the pathological features of adjuvant radiotherapy has a high net clinical benefit in the large threshold probability interval (training set: 0.05–0.90, validation set: 0.30–0.75). This is much higher than that of “treat none” (no pathological features of adjuvant radiotherapy) and “treat all” (all pathological features of adjuvant radiotherapy) groups, as well as Clinic_Sig. All the four models’ efficiency to predict the pathological features of adjuvant radiotherapy is displayed in the Supplementary Table 3.

**Table 2:** Delong test of different signatures

	Clinic_Sig vs. Rad_Sig	Clinic_Sig vs. DTL_Sig	Clinic_Sig vs. DLR_Sig	Rad_Sig vs. DTL_Sig	Rad_Sig vs. DLR_Sig	DTL_Sig vs. DLR_Sig
Training Cohort	< 0.05	< 0.05	< 0.05	0.07	< 0.05	< 0.05
Test Cohort	< 0.05	< 0.05	< 0.05	0.51	0.07	0.72

## Discussion

In our study, we first conducted a screening of the factors pertinent to the pathological features of adjuvant radiotherapy. Subsequently, we employed a machine-learning approach called MLP, to develop a clinical predictive model.Our study revealed a correlation between SCC-AG, MTD-MRI, and the presence of positive lymph nodes on MRI as risk factors. SCC-Ag serves as an early tumor marker for clinical staging and enables monitoring of treatment responses in case of relapse [24]. The serum level of SCC-Ag proves useful in predicting optimal strategies for postoperative adjuvant therapy [25, 26]. Additionally, it exhibits a close correlation with lymph node status [27]. MRI is the optimal imaging modality for assessing tumor size, involvement of vaginal and peripheral tissues [28, 29], interstitial infiltration, and lymph node metastasis in cervical cancer [30]. Implementing a preoperative MRI-based classification strategy reduces the necessity of triple therapy for stage IB cervical cancer [31]. However, the Clinic_Sig, which consists of SCC-AG, tumor size, and positive lymph nodes on MRI, exhibited subpar performance (AUC of 0.64 in the training cohort and 0.53 in the test cohort) in accurately predicting radiotherapy options for ESCSCC.

The T2-weighted sequences are considered the most effective for detecting cervical tumors and their extension to the uterus, parametria, and adjacent organs. Meanwhile, the axial T1-weighted sequence is useful for detecting suspicious pelvic and abdominal lymph nodes. The European Society of Urogenital Radiology (ESUR) recommends an optimized MRI protocol for staging uterine cervical cancer, which includes both T2-weighted and T1-weighted sequences [32]. Next, We selected three MRI sequences (T2WI, T2fs,CE-T1) for acquiring magnetic resonance images. We used MLP to establish the Rad_Sig to analyze intratumoral and peritumoral multi-sequence MRI image, Resnet101 for DRL_Sig to analyze within peritumoral multi-sequence MRI image.In order to evaluate pathological features of adjuvant radiotherapy for ESCSCC management, we integrated Rad_Sig and DRL_Sig by stacking them, resulting in the final prediction termed DTL_Sig. Currently, numerous studies involve the utilization of MRI or other imaging techniques to predict postoperative risk factors in early-stage cervical cancer using traditional radiomics or deep learning models. Liu Y [14] obtained notable outcomes by employing the LASSO method to extract radiomic features from MRI images of T2/SPAIR and CE T1WI. Furthermore, they utilized a radiomic signature to discriminate between adenocarcinoma and squamous cell carcinoma, as well as high-to-moderately and poorly differentiated tumors. Wu Q et al. [15] utilized multiparametric MRI radiomics to evaluate tumor grade, LVSI, and LNM, yielding favorable outcomes. Preoperative MRI-based radiomics exhibited strong predictive performance for LNM in patients with cervical cancer [16, 20], even in cases where pelvic lymph nodes appeared normal in size [19]. Compared to radiologists, radiomics signature based on MRI demonstrates superior preoperative diagnostic capabilities for DOI in early cervical cancer [17]. The multiparametric radiomic features extracted from preoperative MR images accurately predicted the presence of LVSI in patients with cervical cancer [18]. Deep learning models based on CT/MRI images accurately predict LNM [33] or vascular space involvement in patients with cervical cancer prior to surgery [34]. The approach we proposed demonstrated excellent predictive performance, as evidenced by the detection of AUC curves, calibration curves, and decision curve analysis. The AUCs values in both the training cohort and the test cohort suggest that DLR_Sig can reliably differentiate between patients who require adjuvant radiotherapy before surgery and those who do not, as it demonstrates a high accuracy and predictive capability. The DeLong test further indicates that the difference in AUC between Clinic_Sig and DLR_Sig is statistically significant, while the Hosmer Lemeshow test showed that DLR_Sig had a good fit with a *p*-value greater than 0.05. The main reason for this is the DLR_Sig, deep-learning-based radiomics of intratumoral and peritumoral MRI images, which provide a large amount of information and greatly improve the accuracy of predicting postoperative pathological features. The three models, Rad_Sig, DTL_Sig, and DLR_Sig, showed a gradual improvement trend in their performance. However, the information complementarity brought about by the application of artificial intelligence methods in image processing is far less significant than that of Radiomics models and clinical models. This may explain why there was no significant statistical difference among the three models despite their gradual improvement trend. In particular, the DCA curve of DLR_Sig demonstrates greater clinical utility for predicting the pathological features for adjuvant radiotherapy. Early-stage cervical cancer with identified risk factors presents a significant likelihood of recurrence following surgery and should be considered for postoperative adjuvant radiotherapy. Currently, there is no mature method to accurately determine whether postoperative adjuvant radiotherapy is needed before surgery. In comparison to previous investigations, this study constitutes the first attempt to investigate the feasibility of a radiomics model based on multimodal MRI images for predicting the pathological features of adjuvant radiotherapy. This innovative approach aims to identify patients with high-risk pathological features for adjuvant radiotherapy, in order to avoid the adverse effects of multiple treatments by directly undergoing curative radiotherapy instead of surgery.

Our research offers the advantage of evaluating both intratumoral and peritumoral MRI images, providing a comprehensive analysis. While most studies have predominantly focused on the intratumoral region, emerging evidence suggests that imaging features of peritumoral regions can provide valuable information regarding outcomes. Intratumoral and peritumoral CT Radiomics enhances the predictive performance in estimating complete pathological response after neoadjuvant chemoradiation in patients with esophageal squamous cell carcinoma with an AUC of 0.852 (95% CI, 0.753–0.951) [35]. Radiomics based on peritumoral and intratumoral MRI demonstrates an AUC of 0.924 − 0.875 when evaluating lymph node metastasis (LNM) in pancreatic cancer, and an AUC of 0.944 − 0.892 for assessing histological grade [36]. Perilesional radiomics features improved the discrimination ability of the radiomics signature in diagnosing prostate cancer [37]. Radiomics, based on intratumoral and peritumoral functional parametric maps obtained from Breast DCE-MRI, demonstrate predictive capabilities for HER-2 and Ki-67 statuses with respective AUC values of 0.713 and 0.749 [38]. Cui L conducted a study where a radiomics nomogram, utilizing MRI features from peritumoral regions and the degree of cellular differentiation, was able to predict LVSI in patients with cervical cancer, yielding an AUCs of 0.788 − 0.711 [39]. Shi J [40] obtained AUCs of 0.891 − 0.804 in preoperative prediction of lymph node metastasis using MRI-based peritumoral radiomics, MR-reported LN status and tumor diameter in early-stage cervical cancer.

Research has demonstrated an inflammatory response around tumors [41], peritumoral lymphatic vessel density [42], Intratumoral CD8 and peritumoral Foxp3 [43] are closely related to the physiology of the cervix, response to treatment, and prognosis. This could be the biological basis for predicting the pathological characteristics of postoperative adjuvant radiotherapy in ESCSCC using peritumoral MRI images.

### Limitation

We encountered the following limitations. Firstly, data was obtained from a single center; The lack of external validation could render the signatures over fit. Thus, studies obtaining data from many centers are required to remedy this. Secondly, previous studies have shown the value of dynamic contrast enhanced (DCE) and diffusion-weighted imaging (DWI) in predicting pathological features of CSCC [15, 44]. However, we used only T2WI, T2fs, and CE-T1 images in axial views in our retrospective study. Prospective multicenter studies with larger samples are therefore required to validate the robustness and reproducibility of the current research. Finally, due to the limited sample with genetic data, we did not provide genomic characteristics in our study. To advance the field, it is advisable to integrate genomic features, radiomics signatures, and clinical characteristics into records in future study.

## Conclusion

DLR_Sig, which is based on intratumoral and peritumoral MRI images, significantly outperforms Clinic_Sig in predicting the pathological features of adjuvant radiotherapy in early-stage cervical squamous cell carcinoma (ESCSCC). This finding may provide valuable guidance for clinicians and patients in selecting appropriate treatment options.

### Electronic supplementary material

Below is the link to the electronic supplementary material.

Supplementary Material 1

Supplementary Material 2

Supplementary Material 3

Supplementary Material 4

## Data Availability

The datasets used and analysed during the current study are available from the corresponding author on reasonable request.

## Abbreviations

MRI: magnetic resonance imaging
ICC: intraclass correlation coefficient
MTD: maximal tumor diameter
cm: centimeter
CI: confidence interval
LASSO: least absolute shrinkage and selection operator
CNN: Convolutional Neural Networks
MLP: multi-layer preception
AUC: area under the curve
DCA: Decision curve analysis
Clinic_Sig: Clinical Signature
Rad_Sig: Radiomics Signature
DTL_Sig: Deep Transformer Learning Signature
DLR_Sig: Deep Learning Radiomic Signature
ESCSCC: early-stage cervical squamous cell carcinoma
MTD: maximal tumor diameter
PR: postoperative radiotherapy
ALP: alkaline phosphatase
LDH: lactate dehydrogenase ALb:albumin
SCC-Ag: squamous cell carcinoma antigen
LMR: lymphocyte/monocyte ratio
LNR: lymphocyte/neutrophil ratio
