# Supplementary figures and images for: Deep-learning-based radiomics of intratumoral and peritumoral MRI images to predict the pathological features of adjuvant radiotherapy in early-stage cervical squamous cell carcinoma
Source: BMC Womens Health. 2024 Mar 19;24:182. doi: 10.1186/s12905-024-03001-6 (PMC10949581; doi:10.1186/s12905-024-03001-6)

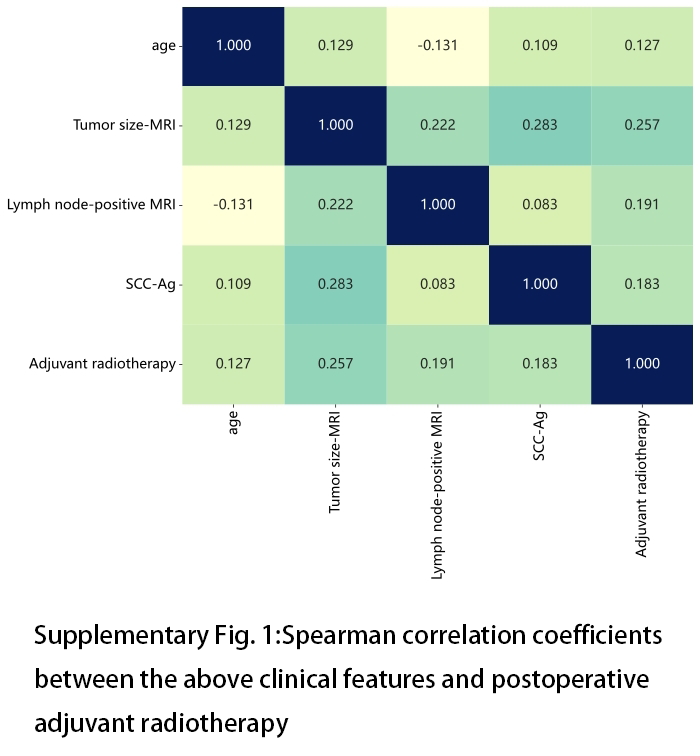

Supplement: Supplementary file 1 — Supplementary Material 1 [file 12905_2024_3001_MOESM1_ESM.tif]

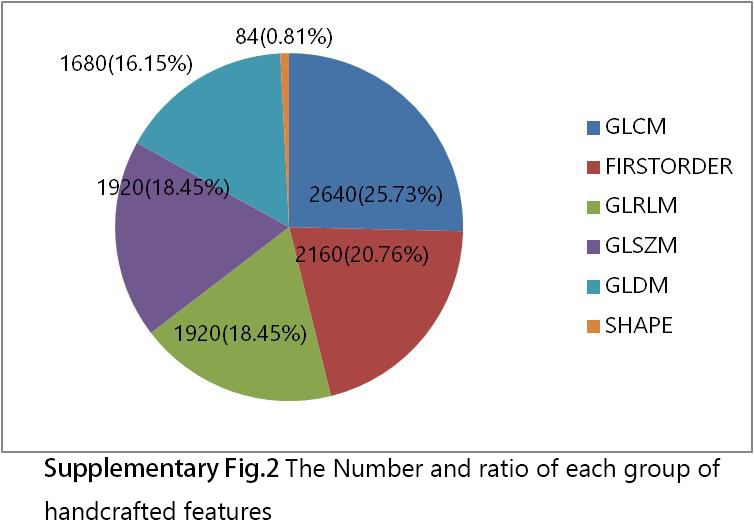

Supplement: Supplementary file 2 — Supplementary Material 2 [file 12905_2024_3001_MOESM2_ESM.tif]

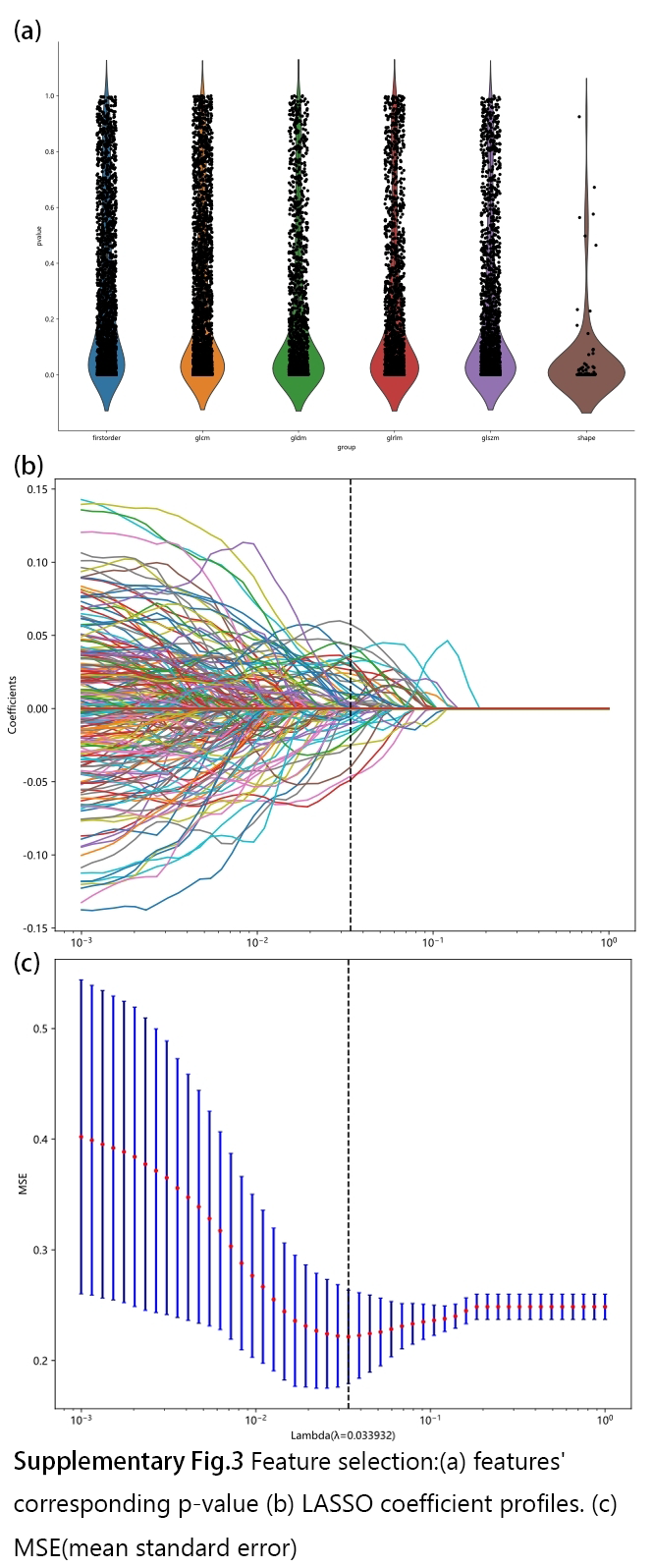

Supplement: Supplementary file 3 — Supplementary Material 3 [file 12905_2024_3001_MOESM3_ESM.tif]
