# Supplementary material for: Deep-learning-based radiomics of intratumoral and peritumoral MRI images to predict the pathological features of adjuvant radiotherapy in early-stage cervical squamous cell carcinoma
Source: BMC Womens Health. 2024 Mar 19;24:182. doi: 10.1186/s12905-024-03001-6 (PMC10949581; doi:10.1186/s12905-024-03001-6)
Supplement: Supplementary file 4 — Supplementary Material 4 [file 12905_2024_3001_MOESM4_ESM.docx]

**Deep-learning-based radiomics of intratumoral and peritumoral MRI images to assess radiotherapy options in early-stage cervical squamous cell carcinoma**

Supplementary material

Supplementary table 1 Clinical characteristics of different MRI scanners (3.0T and 1.5T)

| Variable | 3.0T MRI scanner | 1.5T MRI scanners | P-value |
| --- | --- | --- | --- |
| age(year ) | 49.96±10.27 | 48.98±10.11 | 0.76 |
| MTR-MRI(cm) | 3.40±1.29 | 3.16±1.46 | 0.08 |
| White blood cell(×10^9^/L) | 9.60±3.60 | 10.10±3.39 | 0.23 |
| Neurtophil(×10^9^/L) | 7.67±3.47 | 8.17±3.38 | 0.24 |
| Lymphocyte(×10^9^/L) | 1.35±0.59 | 1.29±0.54 | 0.32 |
| Erythrocytes(×10^12^/L) | 3.67±0.69 | 3.70±0.65 | 0.93 |
| Hemoglobin(G/L) | 104.56±19.59 | 104.74±17.93 | 0.80 |
| Thrombocyte(×10^9^/L) | 226.97±80.90 | 217.56±64.88 | 0.29 |
| ALP(U/L) | 47.88±40.16 | 56.56±38.36 | 0.07 |
| LDH(U/L) | 109.73±88.63 | 127.17±78.85 | 0.03 |
| ALb(G/L) | 24.53±18.05 | 29.85±17.44 | 0.00 |
| Creatinine(mmol/L) | 35.37±31.69 | 28.37±28.31 | 0.04 |
| SCC-Ag(ng/mL) | 3.97±6.63 | 3.21±6.45 | 0.07 |
| Mononuclear cell(×10^9^/L) | 0.51±0.22 | 0.52±0.31 | 0.80 |
| LMR | 3.08±1.97 | 2.91±1.57 | 0.88 |
| LNR | 0.25±0.22 | 0.22±0.19 | 0.18 |
| vagina-positive MRI |  |  | 0.89 |
| No | 58(62.37) | 119(60.71) |  |
| Yes | 35(37.63) | 77(39.29) |  |
| clinical palpation_parametrial_invasion |  |  | 0.58 |
| No | 86(92.47) | 186(94.90) |  |
| Yes | 7(7.53) | 10(5.10) |  |
| Lymph node-positive MRI |  |  | 1.00 |
| No | 79(84.95) | 167(85.20) |  |
| Yes | 14(15.05) | 29(14.80) |  |

MTD-MRI:maximal tumor diameter on MR images of the largest lesions; ALP: alkaline phosphatase; LDH: lactate dehydrogenase ALb: albumin; SCC-Ag: squamous cell carcinoma antigen; LMR: lymphocyte/monocyte ratio; LNR: lymphocyte/neutrophil ratio

Supplementary table 2 Prediction performance of four pre-trained Convolutional Neural Network (CNN) models for the DTL_Sig

| ModelName | AUC | 95% CI | Sensitivity | Specificity | MRI Modal | Cohort |
| --- | --- | --- | --- | --- | --- | --- |
| resnet50 | 0.567936 | 0.4935-0.6424 | 0.792308 | 0.336634 | CE-T1 | Training cohort |
| resnet50 | 0.556364 | 0.4053-0.7074 | 0.272727 | 0.920000 | CE-T1 | Test cohort |
| resnet50 | 0.802323 | 0.7463-0.8584 | 0.669231 | 0.792079 | T2WI | Training cohort |
| resnet50 | 0.642424 | 0.4923-0.7925 | 0.939394 | 0.400000 | T2WI | Test cohort |
| resnet50 | 0.712681 | 0.6473-0.7781 | 0.653846 | 0.683168 | T2fs | Training cohort |
| resnet50 | 0.533333 | 0.3725-0.6941 | 0.939394 | 0.320000 | T2fs | Test cohort |
| *resnet101* | *0.710777* | *0.6447-0.7769* | *0.561538* | *0.792079* | *CE-T1* | *Training cohort* |
| *resnet101* | *0.559394* | *0.4054-0.7134* | *0.757576* | *0.458333* | *CE-T1* | *Test cohort* |
| *resnet101* | *0.813138* | *0.7579-0.8684* | *0.592308* | *0.910891* | *T2WI* | *Training cohort* |
| *resnet101* | *0.763636* | *0.6365-0.8907* | *0.787879* | *0.680000* | *T2WI* | *Test cohort* |
| *resnet101* | *0.800038* | *0.7435-0.8566* | *0.838462* | *0.623762* | *T2fs* | *Training cohort* |
| *resnet101* | *0.689697* | *0.5459-0.8335* | *0.696970* | *0.708333* | *T2fs* | *Test cohort* |
| inception_v3 | 0.571439 | 0.4967-0.6462 | 0.738462 | 0.410000 | CE-T1 | Training cohort |
| inception_v3 | 0.584242 | 0.4352-0.7333 | 0.454545 | 0.720000 | CE-T1 | Test cohort |
| inception_v3 | 0.660168 | 0.5905-0.7298 | 0.746154 | 0.514851 | T2WI | Training cohort |
| inception_v3 | 0.759394 | 0.6338-0.8850 | 0.787879 | 0.640000 | T2WI | Test cohort |
| inception_v3 | 0.760701 | 0.7000-0.8214 | 0.576923 | 0.841584 | T2fs | Training cohort |
| inception_v3 | 0.661212 | 0.5148-0.8076 | 0.878788 | 0.480000 | T2fs | Test cohort |
| densenet121 | 0.627456 | 0.5558-0.6991 | 0.446154 | 0.752475 | CE-T1 | Training cohort |
| densenet121 | 0.555152 | 0.4024-0.7079 | 0.424242 | 0.791667 | CE-T1 | Test cohort |
| densenet121 | 0.781874 | 0.7232-0.8406 | 0.723077 | 0.712871 | T2WI | Training cohort |
| densenet121 | 0.763030 | 0.6375-0.8886 | 0.878788 | 0.560000 | T2WI | Test cohort |
| densenet121 | 0.562338 | 0.4880-0.6367 | 0.353846 | 0.792079 | T2fs | Training cohort |
| densenet121 | 0.632727 | 0.4873-0.7782 | 0.696970 | 0.560000 | T2fs | Test cohort |

Supplementary table 3 Postoperative radiotherapy prediction performance of deep_learning models different sequences

| Modal of different sequences | Cohort | AUC(95% CI) | Sensitivity | Specificity |
| --- | --- | --- | --- | --- |
| CE-T1 | Training cohort | 0.71(0.64-0.78) | 0.56 | 0.79 |
| CE-T1 | Test cohort | 0.56(0.41-0.71) | 0.76 | 0.46 |
| T2WI | Training cohort | 0.81(0.76-0.87) | 0.59 | 0.91 |
| T2WI | Test cohort | 0.76(0.64-0.89) | 0.79 | 0.68 |
| T2fs | Training cohort | 0.80(0.74-0.86) | 0.84 | 0.62 |
| T2fs | Test cohort | 0.69(0.55-0.83) | 0.70 | 0.71 |

AUC: Area Under the Curve; CI: Confidence Interval

Supplementary Table 4. Different models’ Efficiency to predict the need of postoperative radiotherapy

| Signature | Cohort | Accuracy | AUC(95% CI) | Sensitivity | Specificity | Precision | Recall | Threshold |
| --- | --- | --- | --- | --- | --- | --- | --- | --- |
| Clinic_Sig | Training cohort | 0.59 | 0.64(0.57- 0.71) | 0.42 | 0.82 | 0.75 | 0.42 | 0.48 |
| Rad_Sig | Training cohort | 0.91 | 0.97(0.95 - 0.99) | 0.95 | 0.87 | 0.90 | 0.95 | 0.47 |
| DTL_Sig | Training cohort | 0.86 | 0.93(0.90- 0.96) | 0.92 | 0.78 | 0.85 | 0.92 | 0.48 |
| DLR_Sig | Training cohort | 0.96 | 0.98(0.97 - 0.99) | 0.98 | 0.92 | 0.94 | 0.98 | 0.46 |
| Clinic_Sig | Test cohort | 0.55 | 0.53(0.38 - 0.68) | 0.24 | 0.96 | 0.89 | 0.24 | 0.60 |
| Rad_Sig | Test cohort | 0.71 | 0.71(0.58 - 0.85) | 0.88 | 0.48 | 0.69 | 0.88 | 0.20 |
| DTL_Sig | Test cohort | 0.69 | 0.77(0.65 - 0.89) | 0.48 | 0.96 | 0.94 | 0.48 | 0.81 |
| DLR_Sig | Test cohort | 0.72 | 0.79(0.67 - 0.90) | 0.73 | 0.72 | 0.77 | 0.73 | 0.56 |

AUC: Area Under the Curve; CI: Confidence Interval


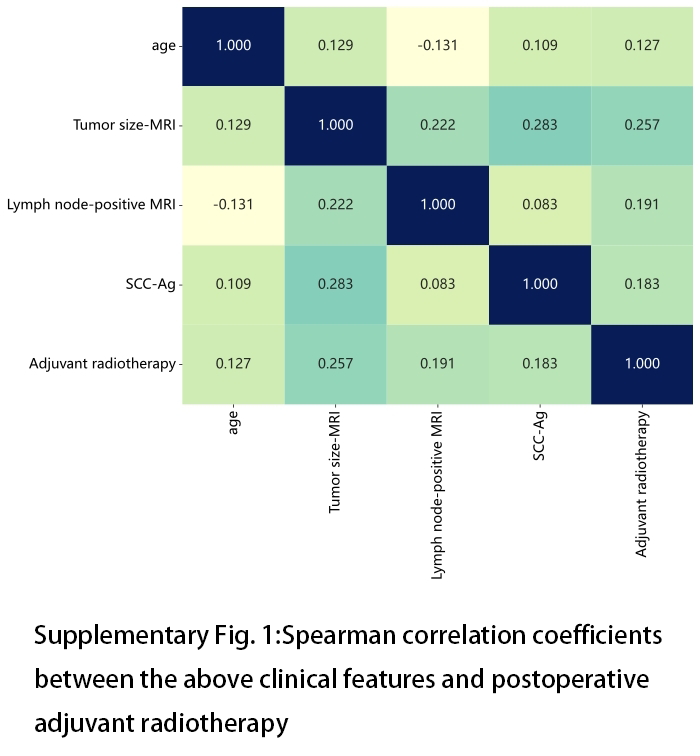


Supplementary Fig. 1:Spearman correlation coefficients between the above clinical features and postoperative adjuvant radiotherapy


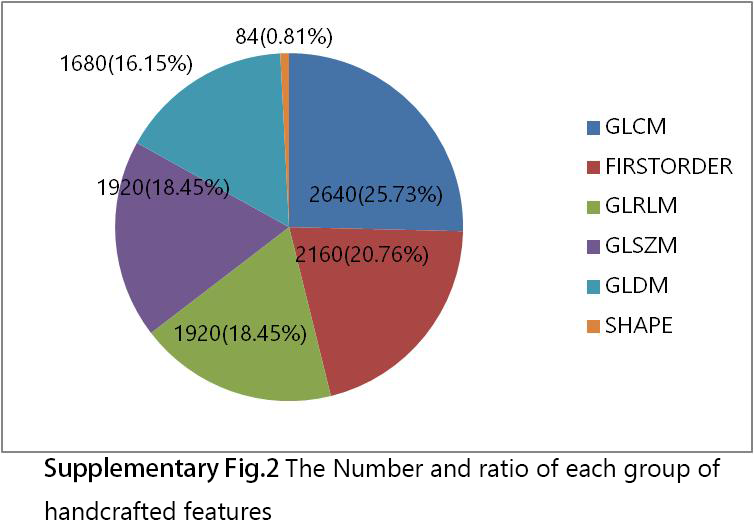


Supplementary Fig.2 The Number and ratio of each group of handcrafted features.


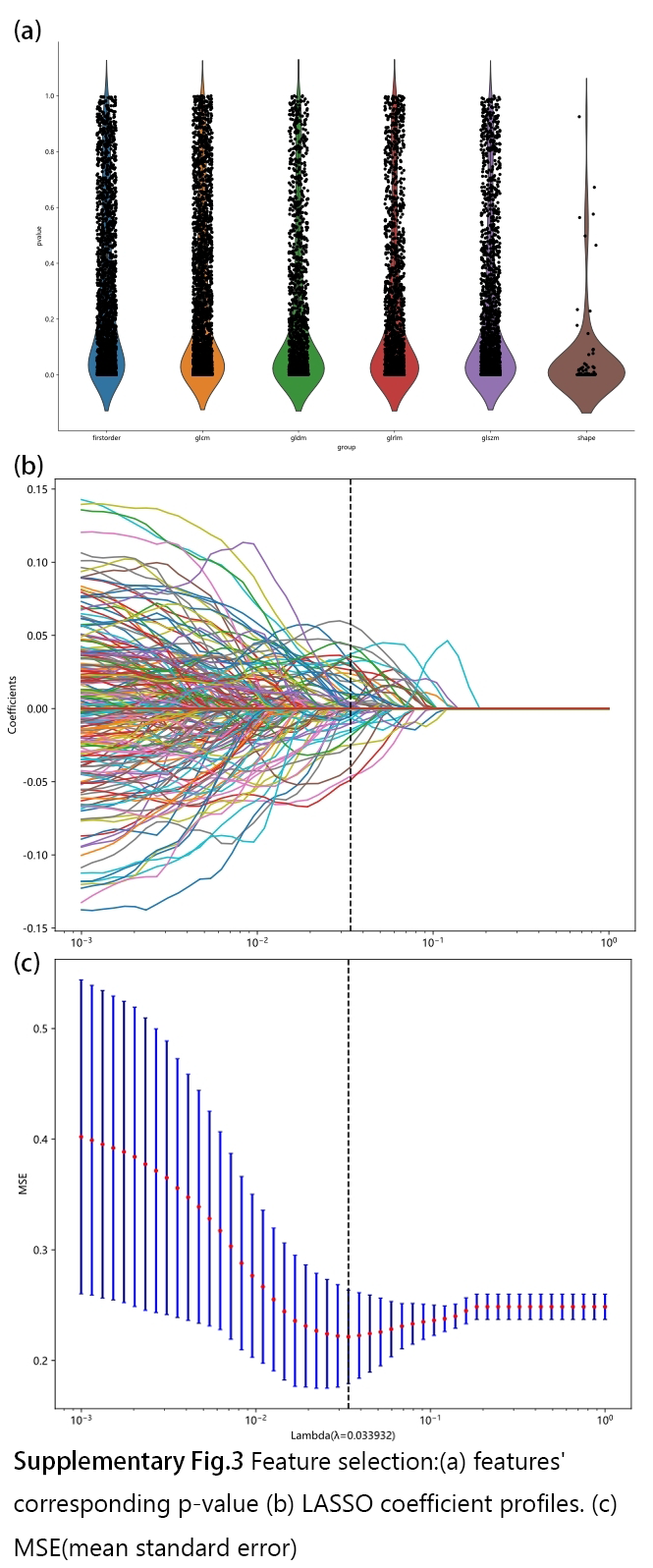


Supplementary Fig.3 Feature selection using the LASSO binary logistic regression model:(a) all features' corresponding p-value results. (b) LASSO coefficient profiles of the features show that are drawn at the value selected using 10 fold cross validation,and the optimal λ results in 49 nonzero coefficients. (c) MSE(mean standard error) of 10 fold cross validation
